# Natural history and outcome in chinese patients with gastroenteropancreatic neuroendocrine tumours: - a 17-year retrospective analysis

**DOI:** 10.1186/s12902-016-0087-9

**Published:** 2016-02-24

**Authors:** Doris T. Chan, Andrea O. Y. Luk, W. Y. So, Alice P. S. Kong, Francis C. C. Chow, Ronald C. W. Ma, Anthony W. I. Lo

**Affiliations:** Department of Medicine and Therapeutics, The Chinese University of Hong Kong, Prince of Wales Hospital, Shatin, Hong Kong; Department of Anatomical and Cellular Pathology, The Chinese University of Hong Kong, Prince of Wales Hospital, Shatin, Hong Kong

**Keywords:** Neuroendocrine tumours, GEP- NETs, Carcinoids

## Abstract

**Background:**

There is rising incidence of gastroenteropancreatic neuroendocrine tumours (GEP- NETs) in many parts of the world, but epidemiological data from Asian populations is rare.

**Methods:**

We conducted a retrospective study in a tertiary medical centre in Hong Kong, using updated diagnostic criteria. The presentation, clinical features, and disease outcome were reviewed for all patients with GEP-NETs confirmed histopathologically at the Prince of Wales Hospital, the Chinese University of Hong Kong, between 1996 and 2013, according to the latest 2010 World Health Organization Classification.

**Results:**

Among 126 patients, GEP- NETs were found in pancreas (34.9 %), rectum (33.3 %), and stomach (8.7 %), and most of them were non- functional GEP- NETs (91.3 %), mostly of grade 1 (G1) (87.3 %), and about 20 % had metastases on presentation. Age under 55 years, G1 tumours and absence of metastases were significant favourable predictors for survival in univariate analysis; whereas G2/3 tumours, size ≥2 cm, and metastases were significant predictors for disease progression (*p* < 0.05). In multivariate analysis, age and metastases on presentation were significant predictors of mortality (respective hazard ratios [HR] 1.05 [95 % confidence interval {CI} 1.02-1.08] and 6.52 [95 % CI 3.22-13.2]) and disease progression (respective HRs 1.05 [95 % CI 1.02-1.07] and 4.12 [95 % CI 1.96-8.68]), while higher tumour grade also independently predicted disease progression (HR 5.17 [95 % CI 2.05-13.05]) (all *p* < 0.05).

**Conclusion:**

Non-functional tumours with non-specific symptoms account for the vast majority of GEP-NETs in this Chinese series. Multidisciplinary approach in the management of patients with GEP-NETs may help improve the treatment efficacy and outcome.

## Background

Neuroendocrine tumours (NETs) refer to tumours originating from neural and endocrine structures distributed throughout the body. They are tumours of the interface between the endocrine and the nervous systems [1]. NETs comprise a heterogeneous family with wide and complex clinical behaviours [2, 3], and they can develop at any sites, with the majority from the gastroenteropancreatic system.

Over the years, the nomenclature and classification of NETs have undergone tremendous changes. In 1907, Oberndofer first described these tumours arising from the epithelial cells in small intestine as “carcinoid”, signifying their relatively indolent growth and “cancer-like” behaviour but not exactly cancers that are more aggressive [4, 5]. It was not until 2000, the term “neuroendocrine tumours” (NETs) was used officially in the WHO Classification, to replace “carcinoid”, which better depicted their malignant potential. In 2010, the WHO classification added a grading system based on the proliferative activity into either G1 (equivalent to carcinoids), G2 or G3 tumours, the latter two were regarded as neuroendocrine carcinomas (NECs).

Most NETs occur in the gastrointestinal tract [6]. In the SEER database (Surveillance Epidemiology and End Results database of the National Cancer Institute) of the United States, there was substantial rise in the overall incidence of gastroenteropancreatic neuroendocrine tumours (GEP- NETs) in the past 30 years, from 1.00 case per 100,000 in the period of 1973–1977, to 3.65 cases per 100,000 in the period of 2003–2007. The statistically significant rise was persistent over the years and was observed across all GEP-NET embryologic subgroups and primary sites [5, 7]. In the SEER database and in many nation-wide cancer registries in other European countries, the increase in overall incidence of GEP-NETs was attributed to the increasing use of abdominal imaging and endoscopy, as well as the inclusion of both benign and malignant GEP-NETs in the registries.

Compared with western countries, there were only a few retrospective studies in Asian countries including Korea, China, Taiwan, India and Malaysia [8–12]. Most of the studies in Asia and the western countries have not used the most updated WHO 2010 Classification, and only very few of them provided data on the long term outcomes. This study aims to provide a detailed analysis of prognosis and outcomes among Chinese patients with GEP-NETs by describing their clinical characteristics, pathological features and clinical outcomes of these patients spanning 16 years at a tertiary endocrine centre in Hong Kong, and to identify the predictors of clinical outcomes.

## Methods

Clinical information for all patients with histologically confirmed gastroenteropancreatic neuroendocrine tumours (GEP-NETs) from the Prince of Wales Hospital, Hong Kong, during the period from January 1996 to August 2013 were identified and included in this analysis. The histological diagnosis and date were retrieved from the Laboratory Information System (LIS) maintained by the Department of Anatomical and Cellular Pathology, Prince of Wales Hospital, Hong Kong. Hand-written and electronic case notes, case summaries and investigation reports of each patient were reviewed to establish patient’s demographic information, details of clinical, biochemical, histopathological and endoscopic or radiological diagnosis of GEP-NETs, subsequent treatment modalities and outcomes. The date of diagnosis was defined as the date of confirmed histological diagnosis of GEP-NETs. For the diagnostic endoscopy or imaging modality, it was defined as the first investigation performed with successful detection of the tumour. Date of progression referred to the date of investigation confirming either local or metastastic progression endoscopically or radiologically. Overall survival was defined as the time from the date of diagnosis to death from all causes in deceased patients, or to the date of last follow-up otherwise. The duration for “progression-free” disease was defined as time of diagnosis to the date of death, or confirmation of regional or distant metastases.

This study was approved by the Joint Chinese University of Hong Kong – New Territories East Cluster Clinical Research Ethics Committee (Reference number: CREC 2013.031), and is in compliance with the Declaration of Helsinki.

### Histological diagnosis, immunohistochemical staining and grading of tumour

The histological slides of each patient were reviewed by one pathologist. Immunohistochemical staining, Ki-67, chromogranin A and synaptophysin staining were performed for all specimens. For pancreatic NETs (pNETs), functional hormonal staining, including gastrin, somatostatin, serotonin, glucagon and insulin, was performed in all specimens. Proliferative indices, Ki-67 and mitotic rate in each specimen were reassessed again to estimate the tumour proliferative activities, and to determine the grade of the tumour according to the WHO 2010 Classification [13]. Tumours with a Ki-67 index of <2 % were classified as G1 tumours, index of 3–20 % were classified as G2, greater than 20 % as G3. Likewise, tumours with mitotic rates of <2/10 HPF were classified as G1, those of 2 to 20/10 HPF were classified as G2, greater than 20/10 HPF as G3. If the grading of Ki-67 index disaccorded with the mitotic rate, the higher one was preferred.

### Statistical analysis

Data is presented as mean ± SD or median (range). Kaplan-Meier and log-rank test were used for univariate analysis of factors including gender, age, primary tumour site (pancreas versus gastro-intestinal-hepatobiliary tract), tumour size, tumour grade according to WHO 2010 classification, chromogranin A immunostaining positivity, functional status of tumour (function versus non- functional tumours), as well as presence of regional lymph node or distant metastasis on presentation. Cox proportional hazard model was used for multivariate analysis of hazard ratio. All statistical tests were two-sided with *p*-value <0.05 being considered as statistically significant. Statistical analysis was performed using the Statistical Package for Social Science Version 18.0 for Windows software package.

## Results

### Patient characteristics, clinical presentation and diagnostic trend

We identified a total of 126 patients diagnosed with GEP-NETs, 64 (50.8 %) of them were male and 62 (49.2 %) were female. The mean age of diagnosis was 56.6 ± 15.2 years old (range 21-98 years old). The most common primary sites were pancreas (34.9 %, n = 44), followed by rectum (33.3 %, *n* = 42), and stomach (8.7 %, *n* = 11). Three (2.4 %) patients had confirmed von Hippel- Lindau syndrome, and one patient had multiple endocrine neoplasia type 1 (MEN1). All of these four patients with associated familial syndromes had non-functional pNETs.

The vast majority of GEP-NETs were non- functional (91.3 %, *n* = 115). Among the 11 functional tumours, insulinoma (81.8 %, *n* = 9) accounted for the majority, while the remaining two tumours included a gastrinoma and an ACTH-secreting pancreatic tumour. As for the non-functioning GEP-NETs, most patients presented with non-specific gastrointestinal symptoms, including epigastric or abdominal pain (33.3 %, *n* = 42), gastrointestinal bleeding (18.3 %, *n* = 23), diarrhoea or change in bowel habit (5.6 %, *n* = 7) and painless progressive jaundice (4.0 %, n = 5). Other manifestations included symptomatic anaemia (4.0 %, *n* = 5) and weight loss (2.4 %, *n* = 3), and one of them presented with pyrexia of unknown origin. About 20 % (*n* = 25) of our patients presented as incidental finding when they had abdominal imaging or endoscopies performed for other purposes such as cancer screening. The remaining four patients were identified in the regular screening of diseases associated with known familial syndromes VHL and MEN1. The distribution and presenting symptoms of patients with GEP-NETs from different sites are detailed in Table 1.

**Table 1:** Distribution of primary tumour sites and corresponding presenting symptoms

Primary tumour site	Patients, N (%)	Male, N	Female, N	Main clinical symptoms
GI Tract	77 (61.1 %)	43	44	
Stomach	11 (8.7 %)	6	5	Abdominal Pain, Gastrointestinal Bleeding, Anaemia
Duodenum	6 (4.8 %)	2	4	Abdominal Pain, Gastrointestinal Bleeding
Ileum	6 (4.8 %)	3	3	Abdominal Pain, Incidental Finding
Appendix	10 (7.9 %)	5	5	Abdominal Pain
Descending Colon	1 (0.8 %)	1	0	Gastrointestinal Bleeding
Sigmoid Colon	1 (0.8 %)	0	1	Abdominal Pain
Rectum	42 (33.3 %)	26	16	Gastrointestinal Bleeding, Incidental Finding
Pancreas	44 (34.9 %)	18	26	Incidental Finding, Abdominal Pain, Hypoglycaemia for Insulinoma
Hepatobiliary System	5 (4.0 %)	3	2	
Liver	4 (3.2 %)	2	2	Abdominal Pain
Cholecystoduodenal Fistula	1 (0.8 %)	1	0	Anaemia

The number of GEP-NETs diagnosed in different time periods from 1996 to 2013 were presented in Table 2. This reflected a substantial increase in the number of pancreatic, rectal and stomach NETs over this time period, which can probably be attributed to the more popular use of abdominal imaging and endoscopy over the past decade. The most common diagnostic procedure was colonoscopy (31.7 %, *n* = 40), followed by oesophago-gastro-duodenoscopy (11.1 %, *n* = 14). Endoscopic ultrasonography (7.9 %, *n* = 10) was increasingly used. On the other hand, computed tomography (CT) scan was the most common initial imaging (19.8 %, *n* = 25), followed by ultrasound (11.9 %, *n* = 15) and magnetic resonance-imaging (MRI) (2.4 %, *n* = 3). A significant proportion were identified as incidental finding during operation for other clinical indications (13.5 %, *n* = 17). The clinical diagnostic information was not available for two of the cases.

**Table 2:** Distribution of GEP- NETs by site across the different periods

Site	1996–2001	2002–2007	2008–2013	Total
Pancreas	11	19	14	44
Rectum	8	17	17	42
Stomach	1	5	5	11
Appendix	3	5	2	10
Ileum	1	4	1	6
Duodenum	0	3	3	6
HBP	2	0	3	5
Colon	0	1	0	1
Sigmoid	0	0	1	1
Total number diagnosed during period	26	54	46	126

### Histopathological characteristics

The median size of the tumours was 1.5 cm (range: 0.1–16.5 cm), of which 87.3 % (*n* = 110) and 98.4 % (*n* = 124) were positive for chromogranin A (CgA) and synaptophysin respectively. The majority of the tumours were G1 (87.3 %, *n* = 110). In addition, 26 patients (20.6 %) had lymph node or distant metastases on presentation and six of them had multiple metastases. The most common sites of metastases were regional lymph nodes and liver (53.8 % each). The most common tumour associated with regional or distant metastases were pancreas (*n* = 8), followed by rectum (*n* = 5), stomach (*n* = 4) and ileum (*n* = 4).

### Clinical outcome

#### Treatment modalities

Most patients underwent curative endoscopic or surgical resections (80.2 %, *n* = 101) whereas 4.0 % (*n* = 5) received operations as palliative measures to relieve tumour-related intestinal or biliary obstructions. Common non-chemotherapy agents administered as adjunct pre-operative medical treatment were diazoxide for insulinomas (*n* = 7) and proton-pump inhibitors (*n* = 1) for gastrinoma. Chemotherapy was used in eight patients, almost always for the purpose of palliative care (*n* = 7). The chemotherapy usually involved combination regimens with platinum-etoposide (*n* = 4), platinum with 5-fluoruracil (*n* = 1), platinum-doxorubicin (*n* = 1), irinotecan capecitabine therapy (*n* = 1) and streptozocin plus 5-fluoruracil (*n* = 1). One patient received platinum-etoposide palliative chemotherapy for recurrent metastatic liver NET and because of lack of response to transcatheter hepatic arterial chemoemobolization (TACE). Palliative radiotherapy was used in two patients with bone metastases to sacrum and right femur. Intra-arterial yttrium-90 microspheres were used in one patient with pNET with liver metastasis. Local-regional therapies such as TACE were used in six patients. Radiofrequency ablation (RFA) was used in one patient with relapse of liver metastasis. Octreotide was used in one patient as pre- operative medical treatment for benign insulinoma. Six patients who presented with metastatic diseases at the time of diagnosis received palliative care.

#### Mortality

One hundred and twenty three patients received long-term follow up with a median duration of 3.23 years (range 0.04–17.15 years). Three of the cases were either lost to follow-up or with their medical case notes unable to be retrieved. The 1-, 3- and 5-year-survival rates were 83.4, 76.0 and 69.3 % respectively. The majority of mortality were related to the tumour (57.9 %, *n* = 22 out of 38).

#### Local/ metastastic relapses

For the 101 patients who received curative surgical or endoscopic resection, 8.9 % (*n* = 9) had local (*n* = 4) or metastatic relapse (*n* = 5), all of which were secondaries to the liver. One patient had local recurrence of insulinoma in the pancreas. The remaining eight patients had their primary tumour in the pancreas (*n* = 4, 50 %), rectum (*n* = 3, 37.5 %) and descending colon (*n* = 1, 12.5 %).

### Prognostic factors for survival and disease progression

Univariate analysis was performed using patients’ age (55 years old as the cut-off point which corresponds to the median age in our study), gender, tumour site (gastrointestinal-biliary tract versus pancreas), tumour grade according to WHO 2010 classification (Grade 1 tumours versus Grade 2 and Grade 3 tumours), size (2 cm as cut-off point), chromogranin A immunostatining positivity, functional status, and presence of metastasis on presentation, to identify potential prognostic factors for survival and disease progression. Age younger than 55 years old (*p* = 0.006), G1 tumours (*p* = 0.001), and absence of metastasis on presentation (*p* < 0.001) were significant predictors of better survival by univariate analysis.

**Fig. 1:**
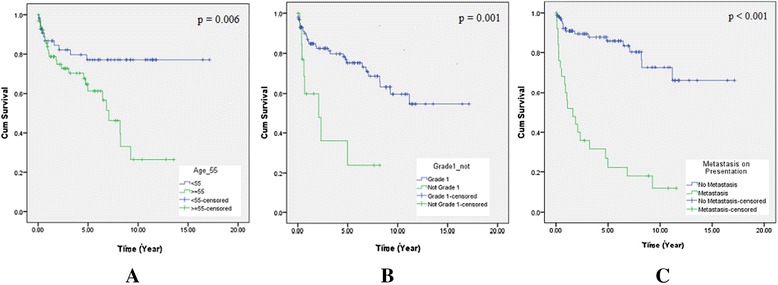
Overall survival. **a** By age - green line indicates age ≥ 55 years old and blue line indicates age < 55 years old (*p* = 0.006). **b** By tumour grade (WHO classification) - green line indicates WHO grade 2 or grade 3 tumours and blue line indicates grade 1 tumours (*p* = 0.001). **c** By metastasis on presentation - green line indicates metastasis on presentation and blue line indicates no metastasis on presentation (*p* < 0.001)

**Fig. 2:**
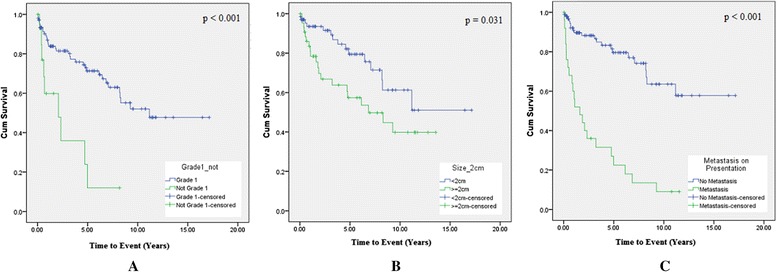
Overall disease progression. **a** By tumour grade (WHO classification) - green line indicates WHO grade 2 or grade 3 tumours and blue line indicates grade 1 tumours (*p* < 0.001). **b** By tumour size - green line indicates tumours ≥ 2 cm and blue line indicates tumours <2 cm (*p* = 0.031). **c** By metastasis on presentation - green line indicates metastasis on presentation and blue line indicates no metastasis on presentation (*p* < 0.001)

For disease progression, tumours grading higher than G1 (*p* < 0.001), tumours larger than or equal to 2 cm (*p* = 0.031), and presence of metastasis on presentation (*p* < 0.001) were significant predictors of disease progression (Table 3). Survival curves and disease progression-free period curves using Kaplan-Meier estimates are displayed in Figs. 1 and 2, respectively. Multivariate analysis using the Cox regression hazard model confirmed that both age and presence of metastasis on presentation were significant independent predictors of mortality and disease progression. Tumour grade was also identified as a significant independent predictor for disease progression (Table 4).

**Table 3:** Overall survival and progression- free disease period

Factors	Overall Survival					Progression- Free Disease Period				
	Number	Mean (Yrs)	95% CI	x^2^	*p*	Number	Mean (Yrs)	95% CI	x^2^	*p*
All Patients	123	10.84	9.25–12.42			123	9.91	8.34-11.48		
Gender				0.122	0.727				0.919	0.338
Male	62	8.04	6.71–9.38			62	7.19	5.84–8.53		
Female	61	11.12	8.90–13.34			61	10.69	8.44–12.94		
Age				7.493	0.006				2.775	0.096
<55	56	13.56	11.68–15.44			56	11.55	9.42–13.69		
≥55	66	7.06	5.47–8.64			66	7.06	5.47–8.64		
Site				2.887	0.089				1.202	0.273
GIH	80	7.94	6.58–9.29			80	7.59	6.24–8.94		
Pancreas	43	12.53	10.14–14.93			43	10.88	8.42-13.35		
Tumour Grading										
G1	106	11.57	9.93–13.22	11.869	0.001	106	10.69	9.04–12.34	15.232	<0.001
G2 or G3	14	3.28	1.37–5.18			14	2.86	1.26–4.45		
Size				2.632	0.105				4.660	0.031
<2cm	67	12.31	10.11–14.51			67	11.82	9.63–14.01		
≥2cm	44	8.61	6.75–10.48			44	7.51	5.68–9.34		
Chromogranin A Staining				0.119	0.730				0.347	0.556
Positive	107	10.86	9.16–12.55			107	10.03	8.35–11.70		
Negative	16	7.81	4.46–11.17			16	6.93	3.71–10.14		
Functionality				2.915	0.088				2.315	0.128
Non- functional	113	8.69	7.48–9.90			113	8.04	6.83–9.24		
Functional	10	15.36	12.24–18.49			10	13.92	10.11–17.73		
Metastases on Presentation				44.760	<0.001				41.259	<0.001
Yes	26	3.39	1.83–4.94			26	3.18	1.74–4.61		
No	96	12.29	11.60–14.98			96	12.23	10.47–13.98

**Table 4:** Cox multivariate analysis for overall survival and disease progression

Mortality			
Variables	HR	95 % CI	*p*
Age	1.05	1.02–1.08	<0.001
Presence of Metastases on Presentation	6.52	3.22–13.19	<0.001
Disease Progression			
Age	1.05	1.02–1.07	0.001
Presence of Metastases on Presentation	4.12	1.96–8.68	<0.001
Tumour Grade	5.17	2.05–13.05	0.001

Variables included in the regression model: age (as continuous variable), tumour size (<2 cm versus ≥2 cm), tumour grade (Grade 1 versus Grade 2 and Grade 3) and status of metastases on presentation (presence of metastases versus no metastasis on presentation). Only significant variables are shown in the table

*HR* hazard ratio, *CI* confidence interval

## Discussion

In this large retrospective analysis of GEP-NETs in Chinese classified according to the latest WHO 2010 Classification, non-functional tumours with non-specific symptoms accounted for the vast majority disease presentation. We also noted significant heterogeneity in the overall prognosis and outcome.

In older Western literature, the small intestine and the appendix were the most common sites of GEP-NETs [5, 14, 15]. Over the past two decades, with increasing availability of endoscopic imaging, rectal NETs have become more common. Among registries in Asia, rectum, pancreas and stomach were the most common primary sites [8–12]. Similarly, rectum (33.3 %) and pancreas (34.9 %) were the most frequently reported sites of GEP-NETs in this analysis, with stomach and appendix together accounting for less than 10 % each. The discrepancy between Western and Asian reports may be due to racial disparities and study design. It should be noted that most of the Western data were derived from population-based studies [5, 14–19], while data in Asia countries were often based on experiences in a single centre or “multiple-centred” studies from limited number of hospitals [8–12]. Nevertheless, this apparent difference in the distribution of GEP-NETs between Asian series and studies reported from the US or Europe may warrant further investigation.

In some series, the Williams and Sandler classification was used [20]. This classification categorizes NETs according to their derivative origins into foregut (lung, stomach, duodenum, proximal jejunum and pancreas), midgut (distal jejunum, ileum, appendix and caecum), and hindgut (transverse and left- sided colon and rectum) [20, 21]. A nationwide epidemiological survey from Japan revealed that hindgut tumours, instead of midgut tumours, were the most common among all GEP- NETs in the Japanese population, with the midgut tumours being the least common [22]. This is in contrast to the Western population [23]. In our study, however, foregut tumours from the pancreas and the stomach accounted for more than one- thirds of all GEP- NETs when the Williams and Sandler classification was adopted. It was thought that this classification by site of origins fails to provide useful pathological and clinical information to prognosticate patients with NETs. It is also imprecise to distinguish different biologically relevant GEP- NETs entities. For example, the foregut tumours which can be from pulmonary, gastric and duodenal, or pancreatic in origin, are too different in their morphology, function and biology to be classified in a single group [24].

Over 90 % of GEP-NETs in our study were non-functional tumours. Of the functional tumours, 82 % were pNETs. The age of diagnosis of functional pNETs was almost 10 years younger than that of non-functional ones, with insulinomas being the most frequently encountered functional tumour. The younger age of presentation is due to the presence of specific symptoms by functional tumours such as recurrent unprovoked hypoglycaemic attacks in patients with insulinomas. While the majority of functional NETs resided in the pancreas, non- functional pNETs nevertheless accounted for the greater proportion. The widespread use of abdominal imaging during work-up for non-specific symptoms may have contributed to the detection of pNETs at an early stage before the development of functional manifestation. Of note, none of our patients presented with carcinoid syndrome, in contrast to the Western populations [12]. Two other single-centred studies in China and Korea also reported low rates of carcinoid syndrome [11, 12]. The reason for this ethnic disparity in the occurrence of carcinoid syndrome is not known.

In this study, regional lymph node and distant metastases occurred in 20.6 %, which is lower compared with the rates (23.0 to 53.4 %) reported by other studies [12, 23]. Most of the GEP- NETs in our review were G1 well-differentiated tumours found incidentally. The lower frequency of small intestine NETs which have greater propensity to metastasize may also account for the relatively lower rate of metastases in our data [12].

In terms of prognostic factors, smaller tumour size, lower tumour grade and absence of metastasis were associated with better survival and less likelihood of disease progression in our study, which concurred with a large retrospective cohort study in Germany [25]. In contrast to results from a single-centre study in Guangdong, China, which demonstrated positive correlation of positive functional status of GEP-NETs with survival in univariate analysis [12], functional status was not found to associate with either disease progression or survival in our analysis. Such discrepancy could be explained by the much smaller number of insulinoma, as well as the overall small proportion of functional tumours in our series.

Endoscopy, USG, CT and MRI remain the most popular diagnostic modalities of GEP-NETs in the current study. In general, the sensitivity of CT and MRI to detect GEP-NETs was estimated to be 28.6 to 94.4 %, and 84 to 95 %, respectively [26]. CT may pick up insulinomas larger than 1 cm in size but not the smaller ones. In this study, biphasic thin section helical CT was performed in all seven patients with insulinoma in whom imaging history was available. Three of them had negative CT findings and the pancreatic tumours were subsequently detected by EUS with the size of insulinomas ranging from 1.2 to 2 cm. The vast majority of NETs are slow-growing with low proliferative index, and express somatostatin receptors, especially subtype 2 (SSTR2) [26, 27]. This forms the basis of somatostatin receptor imaging (SRI) and the rationale for treatments including somatostatin analogue and peptide-receptor radionuclide therapy (PRRT) [27]. ^111^In-DTPA-octreotide is currently the most commonly used radioactive-ligand for somatostatin receptor scintigraphy [27, 28]. The detection rate of ^111^In-DTPA-octreotide scintigraphy is better for gastrointestinal NETs with sensitivity ranging from 80 to 100 % [28], but lower for insulinomas with sensitivity 20 to 60 % [26], as not all insulinomas express SSTR2 [29]. The only three patients with insulinoma in this study who had ^111^In- DTPA scan performed all had negative findings, and the pancreatic lesions were detected using CT or MRI. There are no known uniform guidelines regarding imagings for GEP- NETs. In general, no single technique is 100 % sensitive and specific, multiple imaging modalities should be considered individually to detect small GEP-NETs [26].

Immunohistochemical staining for synaptophysin (Syn) and chromogranin A (CgA) is regarded as part of the standardized pathological assessment of GEP-NETs [30]. Being part of the membrane of neurosecretory hormone granules, positive staining of CgA is strongly dependent on the number of neurosecretory vesicles per cell [13]. CgA is more frequently elevated in well-differentiated tumours compared to poorly differentiated tumours. In our study, CgA was positively stained in immunohistochemical staining in 87.3 % (*n* = 110) of patients. In the remaining 16 CgA-negative patients, however, only two were poorly- differentiated neuroendocrine carcinomas. Synaptophysin (Syn) is a peptide of the small synaptic vesicles present in all neuroendocrine cells [31]. It can be demonstrated in all NETs. In our study, synaptophysin staining was positive in up to 98.4 % (*n* = 124 out of 126), consistent with the high sensitivity of Syn in diagnosis of NETs. Interestingly, immunohistochemical staining for insulin was positive in only six of the nine patients with insulinoma in this study. The negative immunoreactivity for insulin may indicate the production of precursor, or pro-insulin by tumour rather than insulin itself. Moreover, insulin molecules themselves or the antigenicity of insulin molecules may be lost during tissue processing and subsequent immunohistochemical staining.

Among the many therapeutic options for GEP-NETs, surgical treatments of both curative and debulking purposes are the mainstay treatment of choice. Most patients in this review underwent curative endoscopic or surgical resections. In patients with liver metastasis, treatment streams include surgery, loco-regional therapy, systemic medical therapy and ablative procedures such as radiofrequency ablation (RFA), and trans-arterial embolization (TAE) or chemoembolization (TACE). The six patients in our study who received RFA (*n* = 1) or TACE (*n* = 5) experienced recurrence of new metastatic liver lesions after RFA or TACE over a duration of 6 to 14 months. There have not been any randomized trials to examine the superiority of one ablative therapy over another [3].

In terms of systemic medical treatment, there has been adjuvant or neo-adjuvant therapies recommended for high grade NETs after surgery. Traditional chemotherapy is recommended for pNETs, metastatic foregut G2 NETs, and in any G3 tumours with or without liver metastases from various primary sites in the GI tract [32]. In general, well-differentiated NETs are resistant to most chemotherapeutic agents because of their slow proliferation. In our study, patients having metastatic NETs from the gut traditional chemotherapy such as platinum-based agents, all had static or progressive disease after several cycles of chemotherapy. For somatostatin analogues (SA) such as octreotide and lanreotide, they have their therapeutic values proven in functional NETs and metastatic G1 NETs in the midgut. The reported ability for SA to achieve disease stabilization is up to 50–60 % in patients with advanced or metastatic well-differentiated NETs [32, 33]. Another trial also confirmed the anti- proliferative activity of lanreotide for well-to- moderately differentiated non-functional GEP-NETs [33]. In this study, none of the patients received SA. Sunitinib and everolimus are both approved targeted therapies for well- differentiated pNETs. Only one patient of our study used sunitinib. He had von-Hippel Lindau disease and recurrent inoperable non-functional pNET. His pancreatic NET remained unchanged in size for almost three years since the commencement of sunitinib. In our locality, cost is the major constraint of using targeted therapy in metastatic pNETs. There is at present no evidence to support the efficacy of sunitinib and everolimus in treating extra- pancreatic NETs.

To the best of our knowledge, this retrospective review on GEP-NETs at a local tertiary centre is one of the first to report on the clinical presentation, pathological characteristics, investigation and treatment modalities, as well as prognostic factors of GEP-NETs in Asia. In our study, the immunohistochemical staining of chromogranin A and synaptophysin, together with the proliferative indices of Ki-67 and mitotic rate, were re-assessed by one pathologist to ensure the completeness and uniformity of the important pathological information of all specimens. In this way, the grading of the tumour according to the WHO Grade Classification 2010 was assessed accurately to facilitate the analysis of the prognostic significance of tumour grade on survival and disease progression. This approach was not possible in the previously published nation-wide registries in western countries, and was not yet adopted in the most single or multi-centre retrospective studies in Asian countries either [8–11].

There are a number of limitations in this single- centred study, with the major one being the comparatively small number of patients, as well as a small number of events especially mortality. Overall survival was adopted as endpoint rather than disease- specific survival because of this. The retrospective nature of the study also revealed the heterogeneity of disease management among different clinicians in the same tertiary centre under the lack of consensus and guidelines of the management of GEP- NETs. Individual preferences in choosing radiological or biochemical tests for disease staging in diagnosis and disease surveillance were potentially significant factors that could affect the analyses of possible prognostic factors contributing to disease- free survival and mortality. Besides, we could only evaluate the actual number of pancreatic, rectal and stomach NETs diagnosed over different time periods from 1996 to 2013 (Table 2) as it was difficult to calculate the overall incidence rate of GEP-NETs which are still overall a rare disease entity. Moreover, only GEP-NETs with confirmed histological diagnosis were included but not those with only radiological diagnosis. This might exclude patients with advanced disease and large tumour sizes, or patients with multiple metastases which was not resectable for histological diagnosis. Hence, a small number of patients with particularly poor prognosis might have been excluded from the study, leading to the reduced event rate related to the tumour. Taking this into consideration, age may be more of a predictor of overall survival rather than disease- specific survival. Given the study limitations, multidisciplinary approach for patients with GEP- NETs, which involves coordinated delivery of care by a team of specialists from related specialties [34], is expected to be the trend for treatment in the foreseeable future. Multidisciplinary practice allows a more uniform yet individualized management plan for each patient based on updated treatment guidelines and trend. From an academic and research point of view, multidisciplinary care also facilitates large- scale epidemiological research to study the disease pattern, and other opportunities for clinical trials of new treatment options in the future.

## Conclusions

This single-centred retrospective review in Hong Kong provides an expedient description of the epidemiology, clinic-pathological features, management and prognostic factors of GEP-NETs in Asians. With the emergence of various diagnostic and treatment modalities, multidisciplinary care is of paramount importance to improve efficacy of treatment, and the clinical outcomes of patients with GEP- NETs. Further understanding of the molecular mechanisms may improve the treatment and prognosis of patients with GEP-NETs.
